# Is seasonal flowering time of *Paphiopedilum* species caused by differences in initial time of floral bud differentiation?

**DOI:** 10.1093/aobpla/plab053

**Published:** 2021-08-20

**Authors:** Jing-Qiu Feng, Qian Xia, Feng-Ping Zhang, Ji-Hua Wang, Shi-Bao Zhang

**Affiliations:** 1 Key Laboratory of Economic Plants and Biotechnology, Yunnan Key Laboratory for Wild Plant Resources, Kunming Institute of Botany, Chinese Academy of Sciences, Kunming 650201, Yunnan, China; 2 University of Chinese Academy of Sciences, Beijing 100049, China; 3 School of Life Sciences, Yunnan University, Kunming 650500, Yunnan, China; 4 College of Traditional Chinese Medicine, Yunnan Key Laboratory of Dai and Yi Medicines, Yunnan University of Chinese Medicine, Kunming 650500, Yunnan, China; 5 Flower Research Institute of Yunnan Academy of Agricultural Sciences, Kunming 650205, Yunnan, China

**Keywords:** Floral bud anatomy, floral bud differentiation, floral bud initiation, flowering regulation, *Paphiopedilum*, seasonal flowering

## Abstract

Members of the genus *Paphiopedilum* are world-famous for their large, colourful flowers, unique floral morphology and long floral lifespan. Most *Paphiopedilum* species bloom in spring or autumn. The control of flowering time is of great significance to the commercial production of floral crops, because it affects the sales and prices of flowers. However, the mechanism that regulates when *Paphiopedilum* species bloom is unclear. In the present study, floral bud initiation and development of *P. micranthum* (spring-flowering species with one flower per stalk), *P. dianthum* (autumn-flowering species with multiple flowers per stalk) and *P. henryanum* (autumn-flowering species with one flower per stalk) were investigated by morphological and anatomical methods. We divided *Paphiopedilum* floral bud differentiation into six phases: the initiation of differentiation, inflorescence primordium differentiation, flower primordium differentiation, sepal primordium differentiation, petal primordium differentiation and column primordium differentiation. We found that the timing of floral bud differentiation for the three species was synchronized when experiencing the same environment, while the period from initiation to flowering largely differed. In addition, initiation of floral bud differentiation in *P. dianthum* was earlier at a warmer environment. The difference in flowering time of three species was mainly caused by the duration of floral bud development, rather than the initiation time. The findings were of great significance for the cultivation and flowering regulation of *Paphiopedilum* species.

## Teaser

Members of the genus *Paphiopedilum* are world-famous for their unique and delicate flowers. Most *Paphiopedilum* species bloom in spring or autumn, while the mechanism that regulates when *Paphiopedilum* species bloom is unclear. In this study, we found that the difference in flowering of *P. dianthum* (autumn-flowering), *P. micranthum* (spring-flowering) and *P. henryanum* (autumn-flowering) was mainly caused by the duration of floral bud development, rather than the initiation time. In addition, initiation of floral bud differentiation in *P. dianthum* was earlier in warmer environments. The findings are of great significance for the cultivation and flowering regulation of *Paphiopedilum* species.

## Introduction

The transition from vegetative to reproductive development is a crucial phase in a plant’s life (Blázquez 2005; Wahl *et al.* 2013). This transition ensures that plants reproduce during a suitable season and enhances adaptations to their growth environments. In addition, flowering time is important for the cultivation and consumption of flower crops. For instance, matching flowering time with peak periods of flower consumption increases the economic value of flowers. Thus, understanding how flowering time is regulated in plants is vital for floriculture.

Floral induction is mediated through a highly complex signalling network that monitors environmental conditions, such as day length, temperature and nutrient availability (Simpson and Dean 2002; Yanovsky and Kay 2003). One important environmental factor that affects flowering time is temperature. A previous study has found that effective temperatures for flower induction in orchids range between 15 and 25 °C (Lopez and Runkle 2005). For example, temperatures between 18 and 20 °C can induce flowering of *Phalaenopsis* hybrids, whereas higher temperatures inhibit flowering (Huang *et al.* 2016). However, research on the effect of temperature on the initiation and duration of floral bud differentiation in orchids is limited.

In Orchidaceae, floral bud differentiation has been investigated in several taxa, including *Cattleya*, *Bletilla striata*, *Cypripedium*, *Phragmipedium*, *Oncidium*, Apostasioideae, *Chloraea*, *Telipogon*, *Phalaenopsis* and *Dendrobium* (Rotor and MacDaniels 1951; Sano *et al.* 1961; Tanaka *et al.* 1986; Kurzweil 1993; Kocyan and Endress 2001; Weng *et al.* 2002; Pabón-Mora and González 2008; Wei *et al.* 2010; Steinfort *et al.* 2012; Wang *et al.* 2019; Zhang *et al.* 2019). In general, the shoot apical meristem begins to differentiate, initiating the development of floral organs after flowering induction is completed. Floral bud differentiation can be divided into various phases. For example, in *Phalaenopsis* hybrids, floral bud differentiation is divided into six phases: initial differentiation, inflorescence primordium differentiation, flower primordium differentiation, sepal primordium differentiation, petal primordium differentiation, column and pollinia differentiation (Wei *et al.* 2010). Unfortunately, the floral bud differentiation and development in *Paphiopedilum* species has scarcely been studied. However, understanding both when flower bud differentiation is initiated, and the duration of this process are important for regulating flowering time.

The genus *Paphiopedilum* (Orchidaceae) is composed of more than 70 species of orchids, well known for their large, colourful flowers, unique floral morphology and long floral lifespan (Liu *et al.* 2009; Zhang *et al.* 2017; De 2020). The majority of *Paphiopedilum* species bloom only once a year, either in spring or autumn; consequently, natural flowering time of these species does not coincide with periods of peak consumption, which influences their economic value (Li *et al.* 2015). Thus, the regulation of flowering time plays a vital role in the cultivation of *Paphiopedilum* species. A previous study has characterized *Paphiopedilum* floral bud differentiation in a spring-flowering species, *P. armeniacum*, and found that this species initiates floral differentiation in May (Pi *et al.* 2009). However, it is still unclear whether floral bud differentiation differs between spring-flowering and autumn-flowering *Paphiopedilum* species, and if so, what factors underlie these differences.

In the present study, we asked whether differences in flowering time between *Paphiopedilum* species were determined by the initiation time of flower bud differentiation or by the duration of this developmental process. To answer this question, we characterized morphological and anatomical changes during floral bud initiation and development in spring-flowering (*P. micranthum*) and autumn-flowering (*P. dianthum*, *P. henryanum*) species. To investigate whether external factors affect the duration of flower bud differentiation, we grew an autumn-flowering *Paphiopedilum* species at three locations with different growth temperatures. To determine whether internal factors affect the duration of flower bud differentiation, we measured proxies of cost of reproduction and resources acquisition capability (e.g. leaf dry mass per unit area). This study will be helpful for the cultivation and the regulation of flowering time in *Paphiopedilum*.

## Materials and Methods

**Figure 1.**
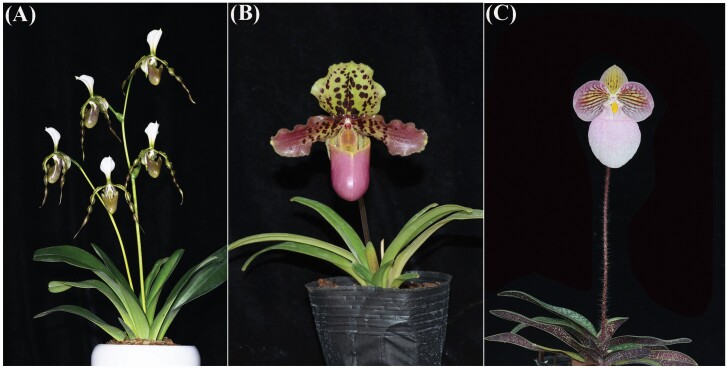
Plant morphology of three *Paphiopedilum* species at the flowering phase. (A) *P. dianthum*; (B) *P. henryanum*; (C) *P. micranthum*.

We examined *Paphiopedilum* flower bud differentiation and development in two autumn-flowering species and one spring-flowering species. *Paphiopedilum dianthum* and *P. henryanum* bloom in autumn. *Paphiopedilum dianthum* has three flowers per stalk on average, whereas *P. henryanum* only has one flower per stalk (Fig. 1A and B). *Paphiopedilum micranthum* blooms in spring and has one flower per stalk (Fig. 1C). All three species occur in limestone areas in south-western China and northern Vietnam (Liu *et al.* 2009), and can be distributed in the same area in Vietnam under natural conditions (Averyanov *et al.* 2003). These three species form new ramets at the base of the plant when they grow to a certain size. Under natural conditions, once individuals mature, new ramets blossom in successive years.

We compared floral bud differentiation and development in three species growing in the same environment. Briefly, 300 mature individuals per species were cultivated in a greenhouse at Kunming Institute of Botany, Yunnan, China (102°44′32″E, 25°08′12″N) at 40 % full sunlight and 50 to 70 % relative humidity. After growing plants 1 month, we collected apical buds from 10 individuals of each species every 15 days until the complete flowers were observed. Samples were preserved in FAA (95 % ethanol:distilled water:formaldehyde:glacial acetic acid, 10:7:2:1, v/v/v/v) for at least 24 h before being dehydrated in an ethanol series and embedded in paraffin for sectioning (Wei *et al.* 2010). Apical buds were sectioned longitudinally (5–8 μm thick) on a Leica RM2126RT rotary microtome (Leica Inc., Bensheim, Germany) and stained with safranine and fast green (Wei *et al.* 2010). The morphology of floral buds was observed and photographed with a light microscope (Leica DM2500, Leica Microsystem Vertrieb GmbH, Wetzlar, Germany), and identified by referring to the study of *Cattleya*, *Cypripedium*, *P. armeniacum*, *Phalaenopsis* (Rotor and MacDaniels 1951; Kurzweil 1993; Pi *et al.* 2009; Wei *et al.* 2010).

We subsequently investigated how temperature affects floral bud differentiation and development in *P. dianthum*. In November 2018, we grew 300 mature individuals of *P. dianthum* at Menglun (23.3 ± 0.2 °C), Puer (17.7 ± 0.2 °C) and Kunming (16.7 ± 0.2 °C), respectively. The temperature was automatically recorded with a HOBO® Pro v2 Logger (Onset Computer Corporation, Bourne, MA, USA). At each site these plants were grown under natural conditions, although shade-nets were used to control the light intensity to less 600 μmol m^−2^ s^−1^. All plants were grown in porous plastic pots (10 cm × 15 cm) filled with bark and humus (7/3, v/v), and watered and fertilized as needed. The morphology of floral bud was examined as previously described.

Peduncle size (PS) was measured with a ruler. The diameter of floral peduncle (PD) was determined with a slide caliper. The leaf number (LN) and flower number (FN) were recorded during anthesis. Individual leaf area (ILA) and individual floral area (IFA) were measured with a Li-Cor 3000A area meter (Li-Cor, Inc., Lincoln, NE, USA). Subsequently, leaves and flowers were oven-dried at 70 °C for 48 h to obtain their dry weights. Leaf dry mass per unit area (LMA) was calculated as leaf dry mass divided by leaf area. Floral dry mass per unit area (FMA) was calculated as floral dry mass divided by floral area. Floral longevity (FL) was the time of individual opening to wilting (Zhang *et al.* 2017). The abbreviations of leaf and flower traits were given in Table 1.

**Table 1.** Leaf and flower traits in the present study.

Traits	Abbreviation	Unit	Significance
Leaf number	LN	no.	Carbon acquisition
Individual leaf area	ILA	cm^2^	Carbon acquisition
Total leaf area	TLA	cm^2^	Carbon acquisition
Leaf dry mass per unit area	LMA	g m^−2^	Leaf cost of light interception
Floral number	FN	no.	Attracting pollinators
Individual floral area	IFA	cm^2^	Attracting pollinators
Total floral area	TFA	cm^2^	Attracting pollinators
Floral dry mass per unit area	FMA	g m^−2^	Floral cost of attracting pollinators
Peduncle size	PS	cm	Mechanical support
Peduncle diameter	PD	mm	Mechanical support
Floral longevity	FL	day	Attracting pollinators

Statistical analysis was performed using SPSS 20.0 for Windows (SPSS Inc., Chicago, IL, USA). A one-way ANOVA was used at *P* = 0.05 significance level to determine the differences in leaf and floral traits among species after testing for normality and homogeneity of variances. All figures were made with image software (Adobe Photoshop 14.0, Adobe Systems Inc., CA, USA).

## Results

### Duration of floral bud differentiation, not initiation time, varied among species

**Figure 2.**
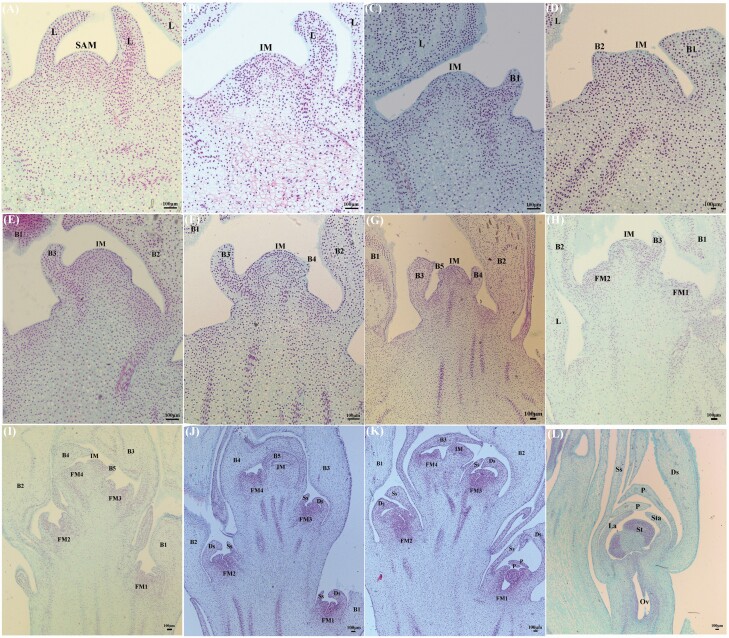
Floral bud differentiation and development in *Paphiopedilum dianthum*. (A) The initiation of differentiation phase. (B–F) The inflorescence primordia differentiation phase. (G) The flower primordia differentiation phase. (H) The sepal primordium differentiation phase. (I and J) The petal primordium differentiation phase. (K and L) The column primordium differentiation phase. L: leaf; SAM: shoot apical meristem; IM: inflorescence meristem; B1: the first bract; B2: the second bract; B3: the third bract; B4: the fourth bract; B5: the fifth bract; FM1: the first flower meristem; FM1: the first flower meristem; FM2: the second flower meristem; FM3: the third flower meristem; FM4: the fourth flower meristem; P: petal; La: labellum; St: stamina; Sta: staminode; Ds: dorsal-sepal; Ss: syn-sepal; Ov: ovary.

**Figure 3.**
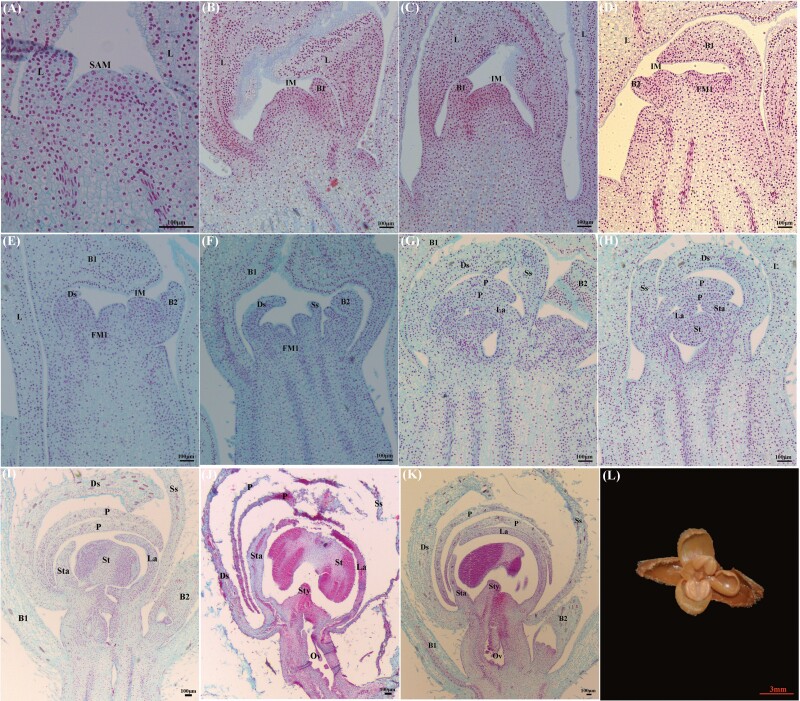
Floral bud differentiation and development in *Paphiopedilum micranthum*. (A) The initiation of differentiation phase. (B and C) The inflorescence primordia differentiation phase. (D) The flower primordia differentiation phase. (E and F) The sepal primordium differentiation phase. (G and H) The petal primordium differentiation phase. (I–L) The column primordium differentiation phase. L: leaf; SAM: shoot apical meristem; IM: inflorescence meristem; B1: the first bract; B2: the second bract; FM1: the first flower meristem; S: sepal; P: petal; La: labellum; St: stamina; Sta: staminode; Ds: dorsal-sepal; Ss: syn-sepal; Sty: style; Ov: ovary.

**Figure 4.**
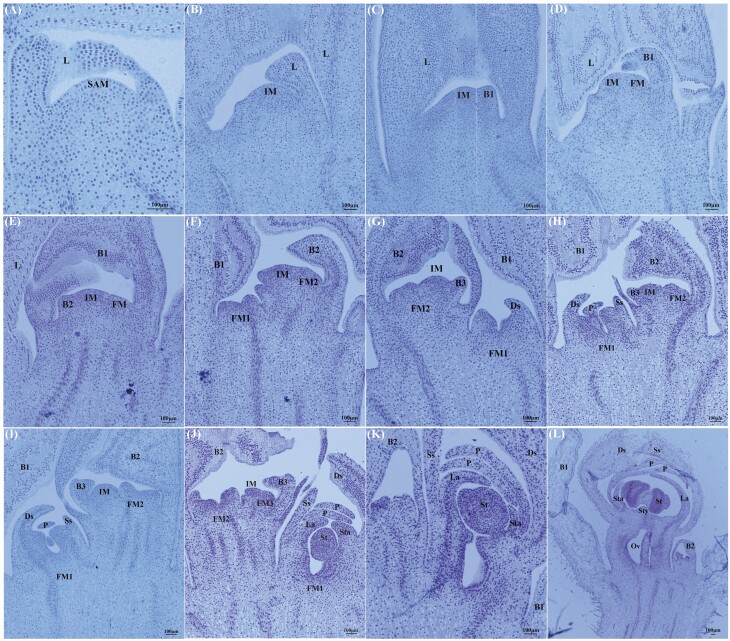
Floral bud differentiation and development in *Paphiopedilum henryanum*. (A) The initiation of differentiation phase. (B and C) The inflorescence primordia differentiation phase. (D and E) The flower primordia differentiation phase. (F and G) The sepal primordium differentiation phase. (H and I) The petal primordium differentiation phase. (J–L) The column primordium differentiation stage. L: leaf; SAM: shoot apical meristem; IM: inflorescence meristem; B: bract; B1: the first bract; B2: the second bract; B3: the third bract; FM1: the first flower meristem; FM2: the second flower meristem; FM3: the third flower meristem; S: sepal; P: petal; La: labellum; St: stamina; Sta: staminode; Ds: dorsal-sepal; Ss: syn-sepal; Sty: style; Ov: ovary.

Floral bud differentiation in *P. dianthum*, *P. micranthum* and *P. henryanum* was divided into six phases: (I) initiation of differentiation, (II) inflorescence primordium differentiation, (III) floral primordium differentiation, (IV) sepal primordium differentiation, (V) petal primordium differentiation and (VI) column primordium differentiation (Figs 2–4). Specifically, the shoot apical meristem is narrow and has high protuberances. During the initiation of differentiation phase (I), the shoot apical meristem has a hemispherical structure encompassed by the leaf (Figs 2A, 3A and 4A). When the shoot apical meristem is transformed into the inflorescence meristem, the hemispherical structure forms flattened protuberances. During the second phase of development (II), bracts differentiate alternately on both sides of the inflorescence axis. In these three *Paphiopedilum* species, the number of bracts vary, i.e. *P. dianthum* (5), *P. micranthum* (2) and *P. henryanum* (2) (Figs 2B–F, 3B and C, 4B and C). As the inflorescence primordium developed, a large space gradually forms under the abaxial bracts. In the phase III, this large space is occupied by small and compacted cells, which display flattened protuberances (Figs 2G, 3D, 4D and E). In the phase IV, the flattened primordium invaginates to form two protuberances. The upper lobe and lower lobes form sepals (Figs 2H, 3E and F, 4F and G). As the sepals grow during phase V, the upper lobe forms the labellum and the lower lobe forms the petal (Figs 2I and F, 3G and H, 4H and I). During petal growth in phase VI, the lower lobe forms the column, which later gradually develops into staminode, stamina, style and stigma (Figs 2K and L, 3I–L, 4J–L).

**Figure 5.**
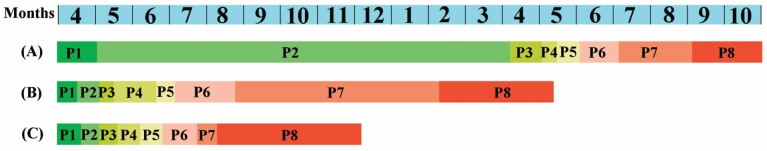
Differences of floral bud differentiation and development for *Paphiopedilum dianthum* (A), *P. micranthum* (B) and *P. henryanum* (C) in Kunming. P1: the initiation of differentiation phase; P2: the inflorescence primordium differentiation phase; P3: the flower primordium differentiation phase; P4: the sepal primordium differentiation phase; P5: the petal primordium differentiation phase; P6: the column primordium differentiation phase; P7: the growth of flower phase; P8: the flowering phase.

The duration of floral bud differentiation, but not initiation time, varied among *Paphiopedilum* species (Fig. 5). An average temperature of 16.9 °C was recorded at a same site in the 2 months before floral bud differentiation. *Paphiopedilum dianthum*, *P. micranthum* and *P. henryanum* initiated floral bud differentiation around May. However, the floral bud differentiation of *P. dianthum* lasted 420 days, with flowers blooming from September to November. In contrast, the duration of flower bud differentiation in *P. micranthum* (270 days) and *P. henryanum* (100 days) was much shorter.

### *Paphiopedilum dianthum* initiated floral bud differentiation and completed differentiation of inflorescence primordium earlier at higher ambient temperatures

**Figure 6.**
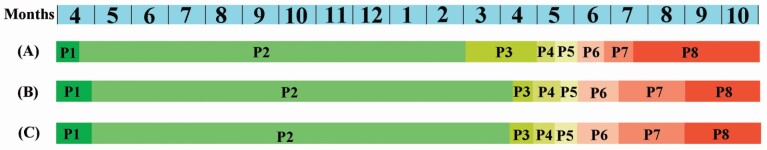
The progress of floral bud differentiation and development in *Paphiopedilum dianthum* at Menglun (A), Puer (B) and Kunming (C). P1: the initiation of differentiation phase; P2: the inflorescence primordium differentiation phase; P3: the flower primordium differentiation phase; P4: the sepal primordium differentiation phase; P5: the petal primordium differentiation phase; P6: the column primordium differentiation phase; P7: the growth of flower phase; P8: the flowering phase.

Although not statistically significant, anatomical observations of *P. dianthum* plants showed that initiation of floral bud differentiation and the differentiation of inflorescence primordium occurred earlier at higher ambient temperatures (Menglun) than at lower ambient temperatures (Puer or Kunming) (Fig. 6). Specifically, *P. dianthum* plants initiated floral bud differentiation about 15 days earlier at Menglun (mid-April, annual average temperature of 23.3 ± 0.2 °C) than at Puer (early May, 17.7 ± 0.2 °C) or Kunming (early May, 16.7 ± 0.2 °C). Similarly, the inflorescence primordium of *P. dianthum* plants began developing about 30 days earlier at Menglun that at Puer or Kunming.

### Differences of leaf and floral functional traits

In our study, the leaf and floral functional traits of *P. dianthum*, *P. henryanum* and *P. micranthum* differed significantly (Table 2). Leaf and flower play vital roles in resources acquisition or maintenance and consumption, respectively. In the resources acquisition, the values of ILA (91.17 ± 5.75 cm^2^), total leaf area (TLA, 397.09 ± 42.66 cm^2^) and LMA (178.80 ± 8.84 g m^−2^) of *P. dianthum* were significantly higher than those of *P. henryanum* (17.35 ± 3.27 cm^2^, 57.75 ± 9.19 cm^2^ and 133.12 ± 6.04 g m^−2^, respectively). The values of ILA and TLA were not significantly different between *P. henryanum* and *P. micranthum*. In the resources consumption, the values of FN, IFA, total floral area (TFA), FMA, PS, PD and FL of *P. dianthum* were higher than those of *P. henryanum*. The values of IFA, TFA and PD of *P. micranthum* were also significantly higher than those of *P. henryanum*, while there were no significant differences in the values of FMA, PS and FL between these two species. The correlation between leaf and flower functional traits showed that TLA was positively correlated with FN, TFA, FMA, PS, PD and FL (Table 3), while PD was positively correlated with FN, TFA, FMA and PS. In addition, FL was also positively correlated with FMA.

**Table 2.** Quantification of floral and leaf functional traits for the three selected *Paphiopedilum* species. LN, leaf number; ILA, individual leaf area; TLA, total leaf area; LMA, leaf dry mass per unit area; FN, floral number; IFA, individual floral area; TFA, total floral area; FMA, floral dry mass per unit area; PS, peduncle size; PD, peduncle diameter; FL, floral longevity; DF, degree of freedom. Different letters indicate significant differences in each parameter among *Paphiopedilum dianthum*, *P. henryanum* and *P. micranthum* (*P* < 0.05, based on ANOVA, followed by Tukey’s *post hoc* tests for comparison).

	Species					
Traits	P. dianthum	P. henryanum	P. micranthum	DF	F-value	P-value
LN (no.)	4.4 ± 0.5^ab^	3.4 ± 0.2^b^	5.0 ± 0.0^a^	2	6.12	0.015
ILA (cm^2^)	91.17 ± 5.75^a^	17.35 ± 3.27^b^	20.94 ± 1.67^b^	2	111.71	<0.001
TLA (cm^2^)	397.09 ± 42.66^a^	57.75 ± 9.19^b^	104.70 ± 8.34^b^	2	51.38	<0.001
LMA (g m^−2^)	178.80 ± 8.84^a^	133.12 ± 6.04^b^	178.87 ± 16.99^a^	2	5.23	0.023
FN (no.)	2.6 ± 0.2^a^	1.0 ± 0.0^b^	1.0 ± 0.0^b^	2	42.67	<0.001
IFA (cm^2^)	52.24 ± 1.56^b^	48.81 ± 1.75^b^	121.77 ± 10.47^a^	2	44.12	<0.001
TFA (cm^2^)	135.98 ± 13.80^a^	48.81 ± 1.75^b^	121.77 ± 10.47^a^	2	21.64	<0.001
FMA (g m^−2^)	53.94 ± 1.90^a^	29.45 ± 1.61^b^	26.23 ± 0.54^b^	2	106.12	<0.001
PS (cm)	38.62 ± 2.49^a^	9.88 ± 0.78^b^	14.50 ± 0.83^b^	2	95.24	<0.001
PD (mm)	4.28 ± 0.14^a^	2.30 ± 0.11^c^	3.22 ± 0.23^b^	2	79.40	<0.001
FL (days)	61.60 ± 1.12^a^	32.60 ± 1.94^b^	29.60 ± 1.50^b^	2	128.71	<0.001

**Table 3.** Pearson correlations between leaf and flower traits for *Paphiopedilum dianthum*, *P. henryanum* and *P. micranthum*. LN, leaf number; ILA, individual leaf area; TLA, total leaf area; LMA, leaf dry mass per unit area; FN, floral number; IFA, individual floral area; TFA, total floral area; FMA, floral dry mass per unit area; PS, peduncle size; PD, peduncle diameter; FL, floral longevity. *P < 0.05; **P < 0.01.

	ILA	TLA	LMA	FN	IFA	TFA	FMA	PS	PD	FL
LN	0.07	0.33	0.35	0.01	0.56*	0.48	0.07	0.23	0.36	0.07
ILA		0.95**	0.37	0.96**	0.37	0.64*	0.94**	0.97**	0.88**	0.95**
TLA			0.36	0.88**	0.28	0.65**	0.93**	0.98**	0.89**	0.93**
LMA				0.40	0.36	0.69**	0.29	0.45	0.59*	0.26
FN					0.40	0.64**	0.87**	0.92**	0.84**	0.89**
IFA						0.44	0.51	0.30	0.06	0.50
TFA							0.43	0.66**	0.78**	0.45
FMA								0.94**	0.82**	0.97**
PS									0.92**	0.93**
PD										0.85**

## Discussion

Our results showed that *Paphiopedilum* species that bloom in spring (*P. micranthum*) initiate floral bud differentiation at the same time as those that bloom in autumn (*P. dianthum* and *P. henryanum*) and that this process can be divided into six phases. However, floral bud differentiation proceeded at significantly different speeds for each species. Thus, differences in flowering time for *Paphiopedilum* species are largely caused by the duration of floral bud development, rather than the initiation time of floral bud differentiation. We also found that the duration of floral bud differentiation is affected by both external (temperature) and internal (e.g. costs of reproduction) factors.

### Floral bud differentiation in three *Paphiopedilum* species

In this study, we divided floral bud differentiation of *Paphiopedilum* species into six phases: initiation of differentiation, inflorescence primordium differentiation, flower primordium differentiation, sepal primordium differentiation, petal primordium differentiation, column primordium differentiation. These findings are consistent with how previous studies have described floral bud differentiation in other orchid species, including *Phalaenopsis* and *Dendrobium* (Wei *et al.* 2010; Zheng *et al.* 2017); however, our description differs from how floral bud differentiation is described in a species such as *Camellia sinensis*, which is divided into five phases (Shi *et al.* 2015).

Floral bud development in *Paphiopedilum* species is consistent with that of species in *Oncidium*, *Phalaenopsis*, *Chloraea* and *Dendrobium*, but not with *Cattleya* (Rotor and MacDaniels 1951; Tanaka *et al.* 1986; Wei *et al.* 2010; Steinfort *et al.* 2012; Zheng *et al.* 2017). In orchids, six tepals of a flower are divided into two whorls. The tepals on the outside become sepals, and the tepals on the inside become petals. Ventral sepals generally fuse into one sepal which is called a syn-sepal; the other sepal is called the dorsal-sepal. One petal is specialized into a labellum. Our characterization of tissue and cellular differentiation in three *Paphiopedilum* species is consistent with that in *Cattleya*, *Phalaenopsis* and *Dendrobium* (Rotor and MacDaniels 1951; Wei *et al.* 2010; Zheng *et al.* 2017). Our anatomical analysis indicated that the bracts emerge first in the inflorescence primordium; the flower primordia invaginates, forming two lobes at the base of bracts next to the axis; the upper lobe develops a syn-sepal and labellum, whereas the lower lobe forms the dorsal-sepal, two lateral petals and the column. In *Cattleya labiate*, in contrast, the two ventral sepals and two lateral petals are derived from the upper lobe (Rotor and MacDaniels 1951). During the growth of floral organ, the ovary or pedicel twists in such a way the inverts the floral bud so that at the time of anthesis the labellum is below the column while the latter is turned away from the axis (Rotor and MacDaniels 1951; Arditti 2005; Chen *et al.* 2009). Similar to the studies mentioned above, our findings indicated that the ovaries of three *Paphiopedilum* species are twisted so that the labellum is in the lower part while the dorsal-sepal is in the upper part of the flower.

### The initiation of floral bud differentiation in *Paphiopedilum* species

The transition from vegetative growth to reproductive growth is a complex process that is determined by various internal and external factors (Wang *et al.* 2019). Floral induction is initiated by temperature and several development pathways, including the age pathway, photoperiod pathway, autonomous pathway, gibberellin pathway and vernalization pathway (Fornara *et al.* 2010; Song *et al.* 2013). Plants cannot bloom in the juvenile phase, unless they reach a threshold size (Pi 2006; Jacquemyn *et al.* 2010). Orchid species usually need 4–7 years of vegetative growth before flowering in the wild (Hew and Yong 2004; Erwin 2007; Wang *et al.* 2010). For example, *P. armeniacum* has similar vegetative growth period with other orchids (Pi 2006).

When grown at the same site, *P. dianthum*, *P. micranthum* and *P. henryanum* all initiate floral bud development in May (Fig. 5). This finding contradicts our hypothesis that species with different flowering time initiate flower bud differentiation at different times. We postulate that floral bud initiation may occur in spring for all *Paphiopedilum* species, and the ambient temperature is the main environmental factors that induce floral bud initiation. Most orchids, including *Phalaenopsis*, are native to tropical areas where the day length does not change dramatically throughout the year (Lopez and Runkle 2005). Thus, it is reasonable to anticipate that photoperiod has limited effect on the flowering of orchids (Wang *et al.* 2019). In contrast, *Phalaenopsis* flowering is known to be promoted by low ambient temperature (Blanchard and Runkle 2006); this induction can be reversed when the ambient temperature is elevated. Low ambient temperatures can also induce flowering activity in *Dendrobium* (Campos and Kerbauy 2004); however, flowering is induced in some *Dendrobium* hybrids by high ambient temperatures (Zheng *et al.* 2017). *Paphiopedilum* species are distributed in tropical and subtropical zones from Asia to the Pacific Islands (Cribb 1998). Consistent with our findings, a previous study has showed that floral bud initiation in *P. armeniacum* occurs in May, during which ambient temperatures are higher than 10 °C, which exceeds the required temperature for vernalization (Pi *et al.* 2009). These results indicate that the environmental requirements for flowering induction of different *Paphiopedilum* species are similar, and the time of floral bud initiation of *Paphiopedilum* species is an adaptation to specific temperature. However, the effect of temperature on flowering induction of *Paphiopedilum* species needs to be confirmed by controlled experiments.

In our study, the initial time of flower bud differentiation for *P. dianthum* was slightly earlier when grown at a higher temperature site (Fig. 6). Our result is consistent with a previous study that demonstrated warm temperature promotes the flowering of *Paphiopedilum* species (Li *et al.* 2015). However, the flowering of *Phalaenopsis* and *Doritaenopsis* species are inhibited by high temperatures (Newton and Runkle 2009). Thus, moderate high temperature may advance the initiation of flower bud differentiation of *Paphiopedilum* species.

### The duration of floral bud differentiation in *Paphiopedilum* species

The present study found that the flowering season, and the time from floral bud initiation to blooming are significantly different among three *Paphiopedilum* species, although their initial times of floral bud differentiation are similar. This indicates that the duration of floral bud differentiation and development is responsible for differences in flowering time.

The duration of floral bud differentiation of *Paphiopedilum* might be affected by internal and external factors. We found that the time of floral bud differentiation and development in *P. dianthum* was affected by temperature, indicating that environmental temperature is an important factor in affecting the flowering time of *Paphiopedilum* species (Fig. 6). This finding is consistent with previous studies that demonstrated floral bud differentiation is influenced by temperature (Li *et al.* 2005; Long *et al.* 2016; Zhang *et al.* 2020). For example, a research has shown that relatively low temperatures can accelerate the flowering of the *Dendrobium* variety ‘Spring Snow’ (Zheng *et al.* 2017). Similarly, temperature has been shown to promote flowering in *Oncidium*, although at high temperature (30 °C) instead of low temperature (Hou and Yang 2009). These results indicate that floral bud differentiation can be accelerated by the appropriate temperature. Increasing winter and spring temperatures often lead to earlier flowering dates in the context of global warming (Miller-Rushing and Primack 2008). The flowering phenology may advance for tropical species according to our study. However, the advance of flowering phenology may be harmful for the survival and reproduction of species due to environmental change and the mismatch between flowering time and pollinator activity in short term. It is necessary to study the flowering phenology by floral bud anatomy by choosing typical species in particular climate area in future.

The duration of floral bud differentiation and development can also be affected by internal factors. Our findings indicate that two internal factors play a major role in the speed of floral bud differentiation in *Paphiopedilum* species. One factor is the resources acquisition and maintenance capability. *Paphiopedilum* species usually distribute in limestone or mountainous forests of tropical and subtropical zones from Asia to the Pacific Islands (Cribb 1998). They usually have thick leathery leaves due to the adaptation to periodic droughts and limited nutrition in their habitats (Averyanov *et al.* 2003). In our study, the LMA value of *P. dianthum* was significantly higher than that of *P. henryanum* (Table 2). Leaf dry mass per unit area (LMA) reflects the leaf construction cost of light capture. Increased LMA usually requires a longer leaf longevity to return the construction cost of the leaf and is accompanied by a longer nutrient residence time. Species with high LMA values often show a conservative resource-use strategy to enable them to increase competitive advantage in water and nutrient-limited habitats (Westoby *et al.* 2002; Guan *et al.* 2011). In addition, we speculate that higher LMA value may explain the more resources needed by *P. dianthum* to maintain their longer longevity because the fleshy leaf is the main storage organ of nutrient and water for *Paphiopedilum* species, but this speculation needs further experimental evidence. In contrast, *P. henryanum* can obtain enough resources in a short time to ensure flowering by leaf photosynthesis (Chang *et al.* 2011).

The second internal factor that affects floral bud differentiation appears to be the cost of reproduction, i.e. developing and maintaining higher numbers of flowers per plant requires more time and resources. Current reproduction of *Spiranthes spiralis* and *Cypripedium acaule* can decrease the flowering and vegetative growth in the next year (Primack and Stacy 1998; Willems and Dorland 2000). This indicates that plants need more time to prepare enough resources to ensure successful reproduction. In our study, *P. dianthum* required over five times as many days as *P. henryanum* to develop complete flowers, and over three times as many days as *P. micranthum* (Fig. 5). *Paphiopedilum micranthum* and *P. henryanum* have one flower per stalk, whereas *P. dianthum* has on average three flowers per stalk, indicating that the cost of developing three flowers per stalk (*P. dianthum*) slows floral bud differentiation (Table 2). The higher FN, PD, PS and FMA values of *P. dianthum* than *P. henryanum* and *P. micranthum* indicate that the former needs more resources to develop its flowers. In addition, the higher FL value of *P. dianthum* than *P. henryanum* and *P. micranthum* indicates that the former also needs more resources to maintain the longer floral lifespan. A previous study has found that FMA is positively correlated with floral longevity, and more resources are needed to maintain flower opening in *Paphiopedilum* species (Zhang *et al.* 2017). The larger PD and TFA values of *P. micranthum* than *P. henryanum* reflect a lager resources consumption in the former than the latter. Thus, the difference in duration of floral bud development among the three *Paphiopedilum* species is correlated with the supply of resources, and the construction and maintenance cost of flowers. Further studies are needed to confirm the difference in the cost of construction and maintenance in *Paphiopedilum* species.

## Conclusions

The present study observed the process of floral bud differentiation in three *Paphiopedilum* species with different flowering time. The initiation time of floral bud differentiation was similar, but the duration of floral bud differentiation and development was different. These findings suggest that the difference in flowering time of three *Paphiopedilum* species is mainly caused by the duration of floral bud development, rather than the initiation time of floral bud differentiation. Growth temperature influenced the differentiation and development of floral bud in *P. dianthum*. Our findings will be helpful for the cultivation and the regulation of flowering time in *Paphiopedilum*. In the future, we will study the effects of temperature and photoperiod on flowering induction and floral bud development of *Paphiopedilum* species.

## Supporting Information

The following additional information is available in the online version of this article—

**supplementary.xlsx**

plab053_suppl_Supplementary_Data

## Data Availability

The raw data are available as **Supporting Information**.

## Notes

**Form & Function. Chief Editor:** Kate McCulloh
